# Protocatechuic Acid-Mediated miR-219a-5p Activation Inhibits the p66shc Oxidant Pathway to Alleviate Alcoholic Liver Injury

**DOI:** 10.1155/2019/3527809

**Published:** 2019-07-24

**Authors:** Rong Fu, Junjun Zhou, Ruiwen Wang, Ruimin Sun, Dongcheng Feng, Zhecheng Wang, Yan Zhao, Li Lv, Xiaofeng Tian, Jihong Yao

**Affiliations:** ^1^Department of Pharmacology, Dalian Medical University, Dalian 116044, China; ^2^Department of General Surgery, The Second Affiliated Hospital of Dalian Medical University, Dalian 116044, China

## Abstract

Alcohol abuse has become common worldwide and has been recognized as a major cause of chronic alcoholic liver disease (ALD). ALD encompasses a complex process that includes a broad scope of hepatic lesions, ranging from steatosis to cirrhosis. In particular, reactive oxygen species (ROS) are mainly involved. Numerous studies have shown that p66shc plays a significant role in ALD. Protocatechuic acid (PCA), a dihydroxybenzoic acid that is naturally found in green tea, vegetables, and fruits, has efficient free radical scavenging effects. In this study, we aimed to assess the protective effect of PCA on ALD and to evaluate the microRNA- (miRNA-) p66shc-mediated reduction of ROS formation in ALD. Our results demonstrated that PCA treatment significantly decreased p66shc expression and downstream ROS formation in ALD. miR-219a-5p, which was identified by bioinformatics and experimental analysis, was enhanced by PCA and subsequently suppressed p66shc expression. Importantly, p66shc played an essential role in the protection of PCA-stimulated miR-219a-5p overexpression. Overall, these findings show that PCA-stimulated miR-219a-5p expression mitigates ALD by reducing p66shc-mediated ROS formation. This study may contribute to the development of therapeutic interventions for ALD.

## 1. Introduction

Alcoholic liver disease (ALD), which is caused by alcohol consumption, has been recognized as a significant risk factor for morbidity and mortality worldwide [1, 2]. ALD encompasses a complex process involving a broad disease spectrum, which includes steatosis, hepatitis, fibrosis, and cirrhosis [3–5]. Also, ROS formation reportedly plays a vital role in regulating ALD [6, 7]. Accordingly, decreasing ROS formation may be effective in preventing and treating alcoholic liver injury.

p66shc, as an isoform of the ShcA adaptor protein family [8], has been suggested to play a pivotal role in inducing oxidative stress, oxidant-induced inflammation, and cell death [9–11]. It has been reported that p66shc regulates ROS formation and causes oxidative cell damage in the liver due to chronic ethanol exposure [12, 13].

MicroRNAs (miRNAs) are short, endogenous, noncoding RNAs that modulate pathophysiological processes [14] and modulate gene expression by binding to complementary sites on target mRNAs [15, 16]. In recent years, studies on p66shc regulation have been in progress, and several miRNAs have been shown to modulate p66shc expression in tissues [17]. Also, miRNAs play predominant roles in many liver diseases, including ALD [18–20]. Despite these observations, whether a miRNA-modulating p66shc is involved in ROS formation in ALD remains unknown.

The expression of p66shc has been remarkably reduced in the lung and intestine by pretreatment with protocatechuic acid, a polyphenolic compound that is commonly present in tea, fruits, and vegetables [21, 22]). In recent years, PCA has been recognized as a chemopreventive compound that possesses biological activities, such as antiatherosclerotic, antioxidant, antiapoptotic, and neuroprotective activities [23, 24]. However, the efficiency of PCA treatment in ALD is currently unclear.

The aims of this study were as follows: (1) to investigate whether PCA prevents ALD by reducing p66shc-mediated ROS formation, (2) to examine the role of miR-219a-5p in regulating p66shc in ALD, and (3) to clarify whether PCA prevents ALD by reducing ROS formation via a miR-219a-5p-p66shc signaling pathway.

## 2. Methods and Materials

### 2.1. Animals and Treatment

Protocatechuic acid (PCA, 98% purity) was purchased from Shanghai Winherb Medical Science Technology Co. Ltd. (Shanghai, China) and dissolved in water. Male Sprague Dawley rats (*n* = 8, 180-220 g) were housed in a special cage with free access to feed and water under controlled conditions at a temperature of 22 ± 2°C, humidity of 60 ± 10%, and a 12 h light/dark cycle. Rats were fed either a normal or a Lieber-DeCarli liquid diet (Dytes, USA) with 5% ethanol (*v*/*v*) for eight weeks. All the liquid diets were freshly prepared before distribution. The control group was pair-fed with an isocaloric liquid diet, where ethanol calories were replaced with maltose-dextrin. During this time, the experimental rats were either not administered PCA or orally administered PCA (10 or 20 mg/kg/d). The dosages of PCA were determined according to previous studies [25, 26], combined with our preliminary animal experiments. Animal models demonstrating the features of chronic ethanol consumption were divided into the following groups: (1) control, (2) control+PCA (20 mg/kg/d), (3) Liber-DeCarli liquid diet, (4) Liber-DeCarli liquid diet+PCA (10 mg/kg/d), and (5) Liber-DeCarli liquid diet+PCA (20 mg/kg/d). Subsequently, liver tissue and blood were harvested for subsequent analysis. The blood samples were centrifuged at 500 g for 15 min, and then serum was collected. All animals received care by the protocols authorized by the Experimental Animal Center of Dalian Medical University (Dalian, China).

### 2.2. Biochemical Analysis

Alanine aminotransferase (ALT, Nanjing Jiancheng Corp., Nanjing, China), aspartate aminotransferase (AST, Nanjing Jiancheng Corp., Nanjing, China), triglyceride (TG, Nanjing Jiancheng Corp., Nanjing, China), and total cholesterol (TC, Nanjing Jiancheng Corp., Nanjing, China) were assayed in serum and liver tissues. Malondialdehyde (MDA), catalase (CAT), glutathione (GSH) levels, and hydrogen peroxide (H_2_O_2_) were determined in liver tissues and cells by using commercial assay kits (Nanjing Jiancheng Corp., Nanjing, China), according to the manufacturer's instructions.

### 2.3. Liver Histological Analysis

Paraffin-embedded liver tissue samples were cut into 5 *μ*m thick sections for hematoxylin and eosin staining (H&E staining), and the sections were then examined by light microscopy.

### 2.4. Immunohistochemistry Analysis

Liver tissues were fixed and embedded in paraffin, and antigen retrieval was constructed by heat mediation in a Tris/EDTA buffer (pH 9). Then, the samples were incubated with a p66shc primary antibody (Abcam, EP332Y, UK). An undiluted HRP-conjugated mouse anti-rabbit IgG antibody was used as the secondary antibody. The liver tissues were counterstained with hematoxylin.

### 2.5. Immunofluorescence Staining

After AML-12 cells were grown in 24-well plates, the cells were fixed with methanol-free 4% formaldehyde for 30 min at room temperature. Then, the cells were washed three times in PBS, blocked with 1% bovine serum albumin in 0.1% Triton X-100, and incubated with an anti-p66shc polyclonal antibody (Proteintech, 10054-1-AP, Wuhan, China) at 4°C overnight. Afterward, the cells were incubated with FITC-conjugated AffiniPure goat anti-rabbit IgG secondary antibodies (Proteintech, FITC-10835, Wuhan, China) for 2 h at room temperature and then counterstained with DAPI for nuclei (Beyotime Institute of Biotechnology, Hangzhou, China). The colors of the images were checked by Vischeck software.

### 2.6. Western Blotting

Western blotting was performed with antibodies against p66shc (BD Pharmingen, 610878, USA), MnSOD (Proteintech, 24127-1-AP, Wuhan, China), and GAPDH (Proteintech, 60004-1-lg, Wuhan, China). Protein quantification was analyzed using ImageJ software (National Institutes of Health, Bethesda, MD) and normalized to GAPDH.

### 2.7. Cell Culture and Treatment

Primary hepatocytes were isolated and cultured according to the previous studies [27]. AML-12 cells, a nontumorigenic mouse hepatocyte cell line (American Type Culture Collection (ATCC), CRT-2254, VA, USA), were cultured in a 1 : 1 mixture of Dulbecco's modified Eagle's medium/Ham's F-12 medium (Gibco, NY, USA) containing 5 *μ*g/ml ITS premix (Sigma-Aldrich, MO, USA), 40 ng/ml dexamethasone (Sigma-Aldrich), and 10% fetal bovine serum (FBS, ScienCell). The cell cultures were maintained at 37°C in a humidified atmosphere with 5% CO_2_. At predetermined time points, cells were pretreated with 10 *μ*M PCA for 6 h, 100 mM ethanol, or neither (control) for 24 h before processing for total protein and RNA extraction. For the experiments, AML-12 cells were transfected with antago-miR-219a-5p (ant-219a, GenePharma, Shanghai, China), ago-miR-219a-5p (ago-219a), or siRNAs using Lipofectamine 3000 (Invitrogen). The sequences of the oligonucleotides are shown in Table 1. After 24 h, the cells were incubated either with or without 10 *μ*M PCA for 6 h and either with or without ethanol for 24 h. Then, the hepatocytes were harvested and processed for total protein and RNA extraction.

### 2.8. Cell Viability

Cell viability was quantitatively analyzed by a Cell Counting Kit-8 (CCK-8) assay (Dojindo, Shanghai, China). Briefly, the experiments were conducted in 96-well plates, and the absorbance of each well was determined at 570 nm.

### 2.9. Quantitative RT-PCR

cDNA was synthesized from total RNA, and reverse transcription was performed with a TaqMan miRNA Reverse Transcription Kit. Mature miRNA was quantified by real-time PCR with a TaqMan miRNA Assay Kit (TransGen Biotech, Beijing, China) using an Applied Biosystems 7300 System (Applied Biosystems, Foster City, CA, USA). miRNA expression was normalized to the endogenous RNA U6 small nuclear 2 (RNU6B) expression.

### 2.10. DCFH-DA Staining

AML-12 cells were incubated with 10 *μ*M DCFH-DA staining (MedChemExpress, NJ, USA) for 30 min at 37°C in a darkroom. DCFH-DA fluorescence was analyzed using a Fluorescence Microplate Reader (Thermo Scientific, IL, USA) with excitation and emission wavelengths of 480 nm and 525 nm, respectively.

### 2.11. Luciferase Activity Assay

AML-12 cells were grown in 24-well plates. Plasmids contained the miR-219a-5p-p66shc 3′ untranslated region (3′-UTR-wt) or the corresponding mutant (3′-UTR-mut) (GenePharma, Shanghai, China). After the plasmid DNA and the ant-219a or the negative controls were cotransfected, AML-12 cells were incubated either with or without 10 *μ*M PCA. Reporter assays were conducted 36 h after transfection. Luciferase activity was determined with a Dual-Luciferase Reporter Assay Kit (TransGen, Beijing, China) using a Dual-Light Chemiluminescent Reporter Gene Assay System (Berthold, Germany) and was normalized to Renilla luciferase activity.

### 2.12. Statistical Analysis

Statistical analysis was performed using GraphPad Prism software (version 5.0; GraphPad Prism Software, La Jolla, CA, USA). All experimental data are reported as the mean ± SD. Statistical analyses were performed using multifactorial one-way analysis of variance (ANOVA) followed by Tukey's post hoc test. The data were analyzed to determine the statistical significance between the groups. Differences with *P* < 0.05 were considered statistically significant.

## 3. Results

### 3.1. PCA Protected Rats against Lieber-DeCarli Diet-Induced Alcoholic Liver Injury

**Figure 1:**
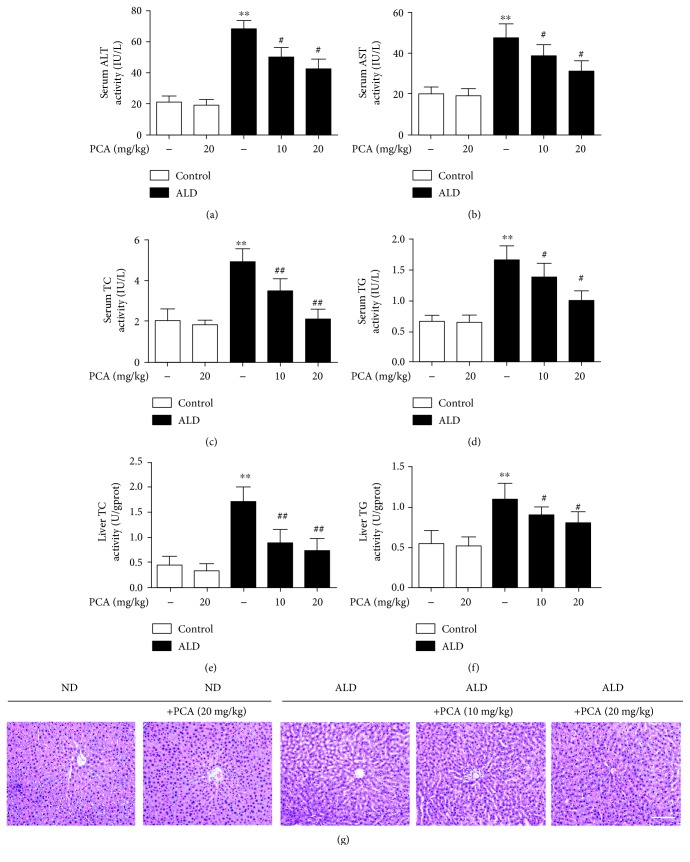
PCA protected rats against Lieber-DeCarli diet-induced alcoholic liver injury. Rats were fed either a normal diet (ND) or a Lieber-DeCarli diet (ALD) alone or in combination with PCA: (a) serum ALT levels, (b) serum AST levels, (c) serum TC levels, (d) serum TG levels, (e) liver TC levels, and (f) liver TG levels; (g) liver tissue sections were stained with H&E. Scale bar = 100 *μ*m. The experimental groups subjected to H&E staining were as follows: ND+PCA, ND+PCA (20 mg/kg), ALD; ALD+PCA (10 mg/kg), and ALD+PCA (20 mg/kg). The data are presented as the mean ± SD (*n* = 10). ^∗∗^ *P* < 0.01 and ^∗^ *P* < 0.05 compared with ND; ^##^ *P* < 0.01 and ^#^ *P* < 0.05 compared with ALD.

To ascertain the effects of PCA on rat liver injury triggered by ALD, we determined the levels of serum ALT, AST, TC, and TG and the levels of liver TC and TG and we conducted H&E staining to confirm the degree of liver injury. Serum ALT, AST, TC, and TG (Figure 1(a)–1(d)) and liver TC and TG (Figures 1(e) and 1(f)) levels were increased in response to alcoholic liver injury, compared with the pair-fed group. These effects were abolished by PCA treatment. Furthermore, PCA treatment attenuated chronic ethanol-exposed structural alterations, according to H&E staining (Figure 1(g)). These data confirmed that PCA protected rats against ALD.

### 3.2. PCA Reduced p66shc-Mediated ROS Formation in Rat Livers and Ethanol-Exposed AML-12 Cells

**Figure 2:**
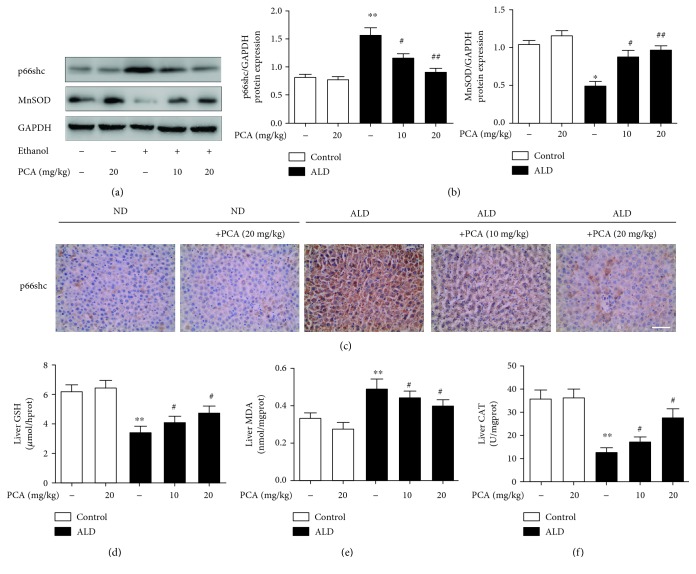
PCA reduced p66shc-mediated ROS formation in rat livers. (a, b) Protein levels of p66shc and MnSOD in rat livers (*n* = 3); (c) immunohistochemistry analysis of p66shc in different groups of rats. Scale bar = 100 *μ*m. (d) Hepatic GSH levels (*n* = 10), (e) hepatic MDA levels (*n* = 10), and (f) hepatic CAT levels (*n* = 10). ^∗∗^ *P* < 0.01 and ^∗^ *P* < 0.05 compared with ND; ^##^ *P* < 0.01 and ^#^ *P* < 0.05 compared with ALD.

Our previous studies and other reports have verified that p66shc expression can be stimulated in ALD [12, 13, 28] and PCA can modulate p66shc expression in different organs [21, 22]. To determine whether PCA treatment could attenuate alcoholic liver injury through p66shc, hepatic p66shc expression was assayed by western blotting and immunohistochemistry (Figure 2(a)–2(c)). The results showed that p66shc expression was increased in ALD and decreased by PCA treatment.

Because p66shc-mediated ROS formation has been confirmed [8, 10, 28], even in chronic alcohol-intake rats [12, 13, 28], we further evaluated the function of PCA on decreasing p66shc-mediated ROS formation in ALD. Rats that consumed alcohol showed decreased levels of MnSOD protein expression (Figures 2(a) and 2(b)) and hepatic GSH and CAT (Figures 2(d) and 2(f)) but displayed high levels of hepatic MDA (Figure 2(e)). In contrast, PCA treatment significantly reversed the decrease in MnSOD expression and GSH and CAT levels and caused the decrease in MDA levels (Figure 2(a)–2(f)).

**Figure 3:**
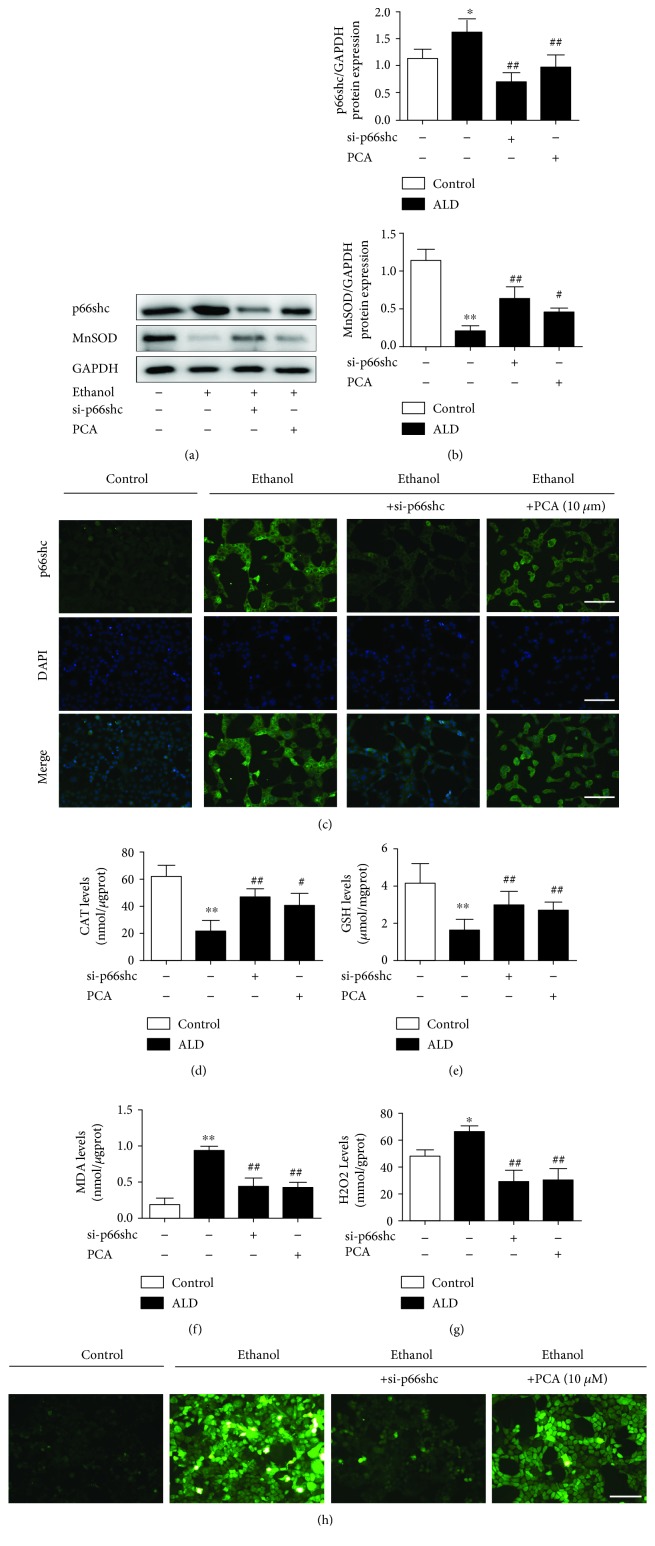
PCA reduced p66shc-mediated ROS formation in ethanol-exposed AML-12 cells. AML-12 cells were transfected with p66shc-specific siRNA or control siRNA for 36 h, 10 *μ*M PCA for 6 h, and/or 100 mM ethanol for 24 h. (a, b) Protein levels of p66shc and MnSOD (*n* = 3); (c) immunofluorescence staining for the p66shc antibody in AML-12 cells for proliferation analysis. Scale bar = 100 *μ*m. (d) Hepatic CAT levels (*n* = 10), (e) hepatic GSH levels (*n* = 10), (f) hepatic MDA levels (*n* = 10), and (g) hepatic H_2_O_2_ levels (*n* = 10). (h) DCFH-DA staining. Scale bar = 100 *μ*m. ^∗∗^ *P* < 0.01 and ^∗^ *P* < 0.05 compared with the control group; ^##^ *P* < 0.01 and ^#^ *P* < 0.05 compared with the ethanol group.

Based on the in vivo findings, AML-12 cells were transfected with si-p66shc, and as anticipated, MnSOD protein expression (Figures 3(a) and 3(b)) and GSH and CAT levels were increased and MDA and H_2_O_2_ levels were decreased (Figure 3(d)–3(g)). DCFH-DA staining, which was used to determine intracellular ROS formation, also showed that the levels of ROS were attenuated when p66shc was silenced (Figure 3(h)). In vitro, 10 *μ*M was selected as the primary concentration of PCA (Supplementary Figure 1). PCA-pretreated cells decreased p66shc expression levels and the ROS formation induced by ethanol exposure (Figure 3).

### 3.3. miR-219a-5p Decreased p66shc Expression in ALD

**Figure 4:**
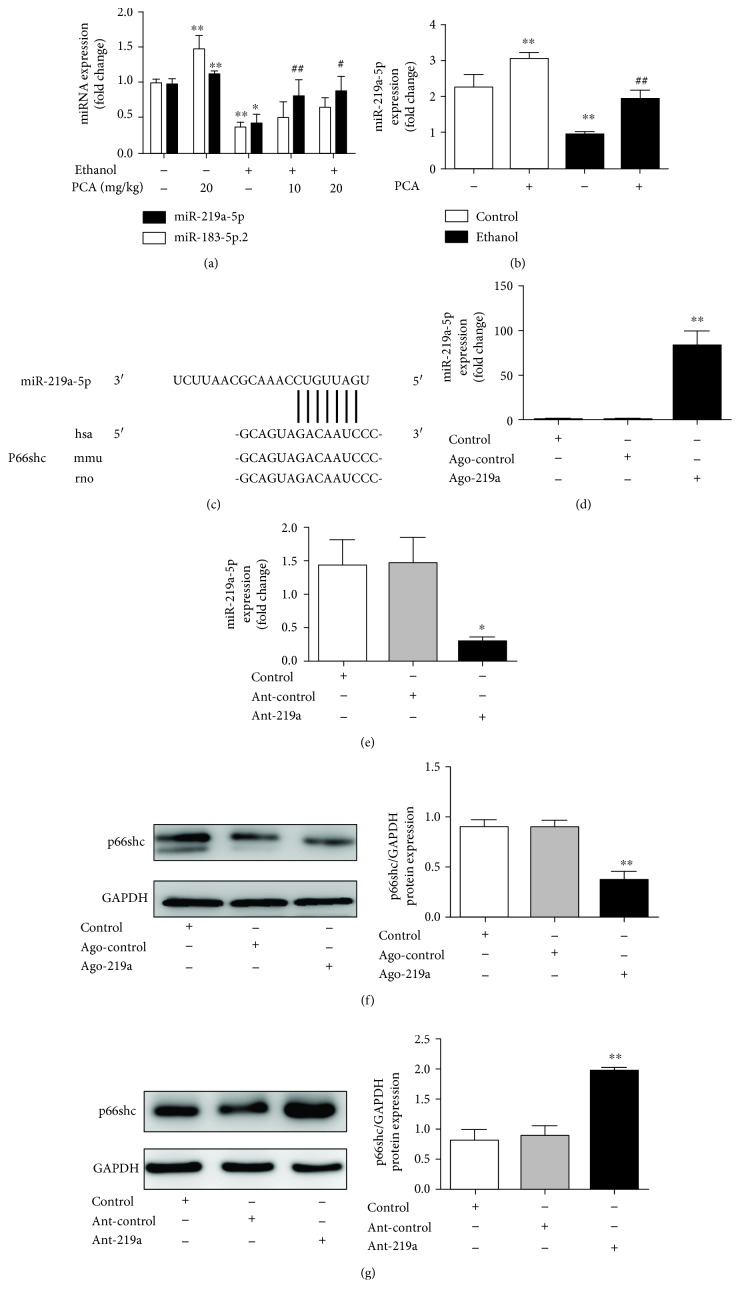
miR-219a-5p decreased the p66shc expression in ALD. (a) qRT-PCR analysis of the miRNA expression in different groups of rats (*n* = 6). ^∗∗^ *P* < 0.01 and ^∗^ *P* < 0.05 compared with ND; ^##^ *P* < 0.01 and ^#^ *P* < 0.05 compared with ALD. (b) qRT-PCR analysis of the miR-219a-5p expression in ethanol-exposed AML-12 cells (*n* = 6). ^∗∗^ *P* < 0.01 and ^∗^ *P* < 0.05 compared with the control group; ^##^ *P* < 0.01 and ^#^ *P* < 0.05 compared with the ethanol group. (c) The miR-219a-5p target sequence in the p66shc 3′-UTR is conserved across various species. (d–g) AML-12 cells were transfected with ago-219a or ant-219a to upregulate or inhibit miR-219a-5p. Ago-NC or antago-NC was used as a negative control. (d, e) qRT-PCR analysis of miR-219a-5p expression (*n* = 6). (f, g) p66shc protein levels (*n* = 3). ^∗∗^ *P* < 0.01 and ^∗^ *P* < 0.05 compared with ago-NC or antago-NC.

It has been reported that miRNA plays an important role in ALD [19, 20, 29], and some studies have shown that p66shc could be regulated by miRNA [17]. Accordingly, we hypothesized that miRNAs might be implicated in the modulation of p66shc in ALD. To investigate the miRNA expression profiles in ALD, we referred to the literature associated with the ALD model [18, 30, 31] (Table 2). The miRNA target prediction program TargetScan (http://www.targetscan.org/) was used to identify miRNAs that target the p66shc 3′ untranslated region (3′-UTR). Among the 10 downregulated miRNAs, miR-183-5p.2 and miR-219a-5p, with high conservation, were predicted to bind to the 3′-UTR of p66shc mRNA. The expression of the two miRNAs was further assayed by qPCR in vivo and in vitro (Figures 4(a) and 4(b)). The in vivo qPCR results indicated that both miRNA expression levels were significantly suppressed in the ALD rats, but miR-183-5p.2 showed no response to PCA pretreatment in ALD (groups 4 and 5, Figure 4(a) and Table 3). Furthermore, miR-183-5p.2 expression was undetectable by qPCR in AML-12 cells exposed to ethanol (Table 4). Compared with the relative miRNAs, ago-219a-5p decreased p66shc expression, while ago-219a-2-3p and ago-219b-5p failed to regulate p66shc expression (Supplementary Figure 3).

Then, we examined whether p66shc could be regulated by miR-219a-5p modulation. AML-12 cells were transfected with ago-219a or ant-219a. Along with marked changes in the miR-219a-5p expression (Figures 4(d) and 4(e)), p66shc protein expression was decreased after ago-219a transfection or increased after ant-219a transfection (Figures 4(f) and 4(g)).

### 3.4. miR-219a-5p Exerted Protective Effects on ALD in a p66shc-Dependent Manner

**Figure 5:**
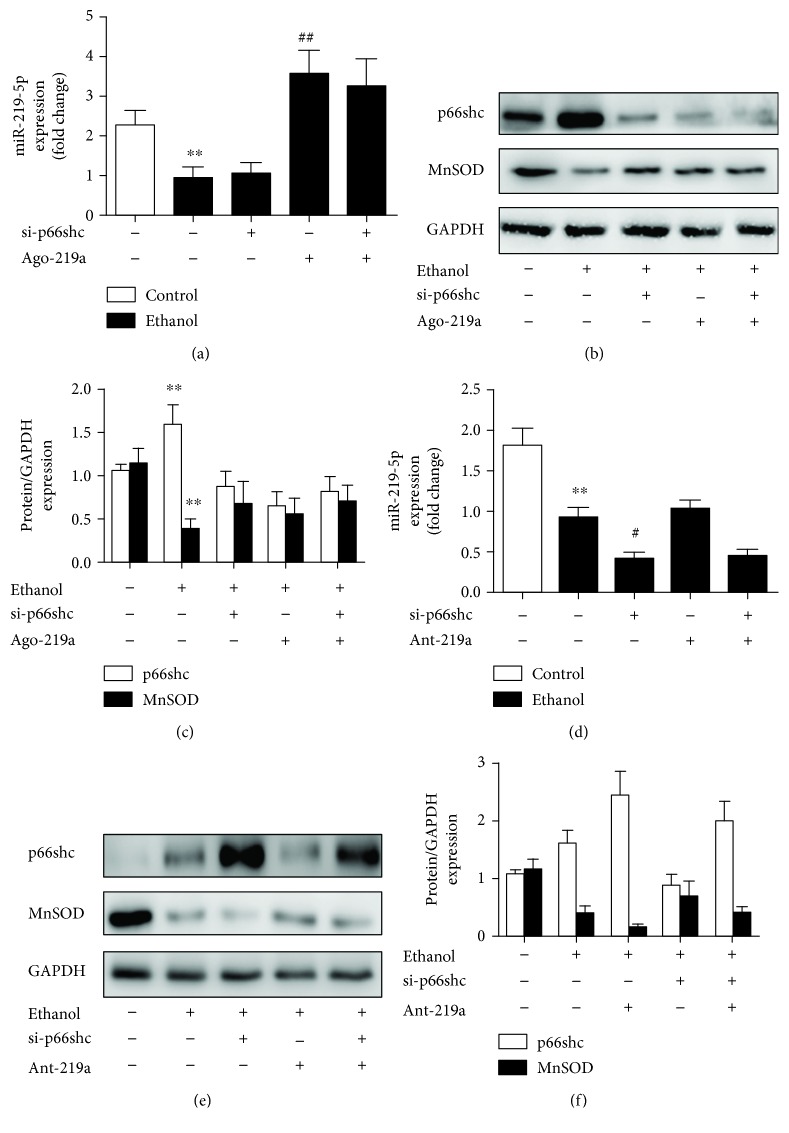
miR-219a-5p attenuated p66shc-mediated ROS formation in vitro. (a–c) AML-12 cells were cotransfected with ago-219a, si-p66shc, or the corresponding negative control, as indicated. (a) qRT-PCR analysis of the miR-219a-5p expression (*n* = 6). (b, c) Protein levels of p66shc and MnSOD (*n* = 3). (d–f) AML-12 cells were cotransfected with ant-219a, si-p66shc, or the corresponding negative control, as indicated. (d) qRT-PCR analysis of the miR-219a-5p expression (*n* = 6). (e, f) Protein levels of p66shc and MnSOD (*n* = 3). ^∗∗^ *P* < 0.01 and ^∗^ *P* < 0.05 compared with the control group; ^##^ *P* < 0.01 and ^#^ *P* < 0.05 compared with the ethanol group; ^&&^ *P* < 0.01 and ^&^ *P* < 0.05 compared with cells transfected with ant-219 with ethanol.

Based on the findings mentioned above, we examined whether miR-219a-5p exerted protection against ALD via p66shc. As shown in Figures 5(a) and 5(d), p66shc silencing had no effect on miR-219a-5p expression in the ethanol-exposed AML-12 cells. Then, we transfected AML-12 cells with si-p66shc and ago-219a either together or apart. As anticipated, ago-219a decreased the p66shc expression and increased the MnSOD expression, but si-p66shc transfected together with ago-219a inhibited the ago-219a-mediated protection (Figure 5(a)–5(c)). Meanwhile, when AML-12 cells were transfected with si-p66shc and ant-219a either together or apart, ant-219a increased the p66shc expression and decreased the MnSOD expression and with p66shc silencing partly alleviated the increase in p66shc and loss in MnSOD (Figure 5(d)–5(f)). Overall, on the basis of p66shc silencing, further modulation of the miR-219a-5p expression on p66shc and MnSOD protein levels, ago-219a, or ant-219a was mitigated compared to the respective transfected group. These results indicate that miR-219a-5p at least partly affects ALD in a p66shc-dependent manner.

### 3.5. PCA Inhibited p66shc Expression by Stimulating miR-219a-5p Expression

**Figure 6:**
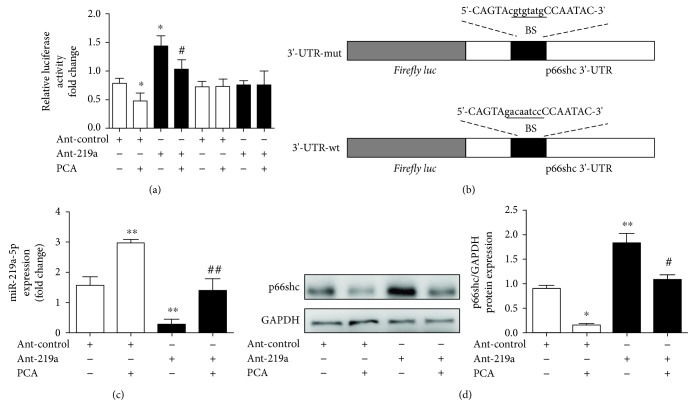
PCA inhibited the p66shc expression via stimulation of miR-219a-5p. (a) Luciferase assay of AML-12 cotransfected with reporter constructs containing p66shc 3′-UTR-wt or 3′-UTR-mut and with ant-219a or antago-NC, as indicated (*n* = 3). (b) Schematic of the wild-type p66shc 3′-UTR (3′-UTR-wt) and mutated p66shc 3′-UTR (3′-UTR-mut) luciferase constructs. BS: binding site. (c, d) AML-12 cells were transfected with either ant-219a or antago-NC for 24 h and 10 *μ*M protocatechuic acid treatment for 6 h. (c) qRT-PCR analysis of the miR-219a-5p expression (*n* = 6). (d) Protein levels of p66shc (*n* = 3). ^∗∗^ *P* < 0.01 and ^∗^ *P* < 0.05 compared with the antago-NC group; ^##^ *P* < 0.01 and ^#^ *P* < 0.05 compared with the ant-219a group.

To further clarify the modulation of the p66shc expression by PCA via miR-219a-5p, we generated a p66shc 3′-UTR luciferase reporter containing miR-219a-5p-binding sites (p66shc wild-type 3′-UTR) or mutated binding sites (p66shc 3′-UTR-mut). The construct was cotransfected into AML-12 cells with ant-219a or the miRNA negative control (antago-control). The results indicated that ant-219a markedly enhanced the luciferase activity induced by the wild-type 3′-UTR of p66shc compared with the antago-control in AML-12 cells (Figure 6(a)). In addition, the luciferase activities of the mutated p66shc 3′-UTR failed to be stimulated by ant-219a (Figure 6(b)).

To further examine the PCA-regulated protective functions of miR-219a-5p on p66shc, we transfected AML-12 cells with ant-219a in either the presence or absence of PCA and measured the expression of miR-219a-5p and p66shc. Consistent with the luciferase assays, the miR-219a-5p expression was substantially decreased after ant-219a transfection compared with the control group, while PCA enhanced miR-219a-5p expression (Figure 6(c)). Furthermore, silencing miR-219a-5p increased the p66shc expression in AML-12 cells (Figure 6(d)). Notably, PCA reversed the increase of p66shc induced by miR-219a-5p silencing. Thus, we concluded that PCA might target the miR-219a-5p/p66shc pathway in AML-12 cells.

### 3.6. PCA Exerted an Antioxidant Effect through the miR-219a-5p/p66shc Pathway with Ethanol Exposure

To further investigate the PCA-mediated miR-219a-5p/p66shc antioxidant signaling on ALD, AML-12 cells were transfected with the miR-219a-5p agomir in either the presence or absence of PCA with ethanol exposure, and the expression levels of p66shc and MnSOD were determined.

**Figure 7:**
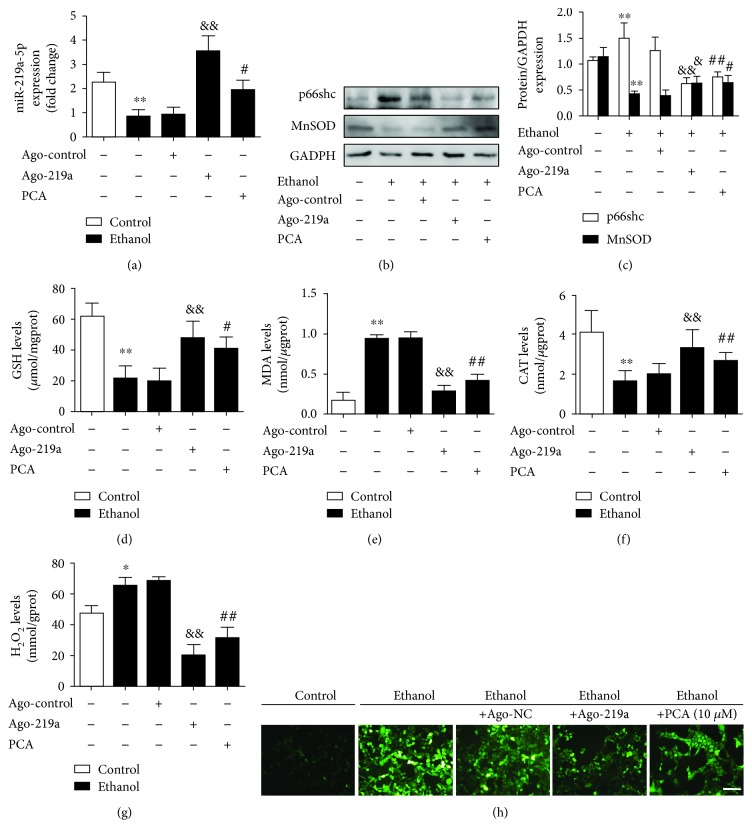
PCA exerted an antioxidant effect through the miR-219a-5p/p66shc pathway with ethanol exposure. AML-12 cells were transfected with antogo-miR-219a-5p or antago-NC for 24 h, treated with 10 *μ*M PCA for 6 h and/or 100 mM ethanol for 24 h. (a) qRT-PCR analysis of miR-219a-5p (*n* = 6). (b, c) Protein levels of p66shc and MnSOD (*n* = 3). (d) Hepatic GSH levels (*n* = 10), (e) hepatic MDA levels (*n* = 10), (f) hepatic CAT levels (*n* = 10), and (g) hepatic H_2_O_2_ levels (*n* = 10). (h) DCFH-DA staining. Scale bar = 100 *μ*m. ^∗∗^ *P* < 0.01 and ^∗^ *P* < 0.05 compared with the control group; ^##^ *P* < 0.01 and ^#^ *P* < 0.05 compared with the ethanol group; ^&&^ *P* < 0.01 and ^&^ *P* < 0.05 compared with cells transfected with ago-NC with ethanol.

PCA and ago-219a significantly inhibited ROS formation in ALD (Figure 7(b)–7(g)). Consistent with the in vivo results, miR-219a-5p was decreased in ethanol-exposed cells, while miR-219a-5p was upregulated with PCA treatment or ago-219a transfection (Figure 7(a)). Correspondingly, the p66shc protein expression and MDA and H_2_O_2_ levels were increased, and MnSOD protein expression levels and GSH and CAT levels were downregulated with ethanol exposure. DCFH-DA staining also indicated that intracellular ROS formation was increased. However, these effects were abrogated by PCA or ago-219a (Figure 7(b)–7(g)). Consequently, it was inferred that PCA might increase the miR-219a-5p expression to inhibit p66shc protein levels and downstream ROS formation in ALD. Meanwhile, ethanol metabolism might be partly linked to the miR-219a-5p/p66shc pathway in ALD (Supplementary Figure 4).

**Figure 8:**
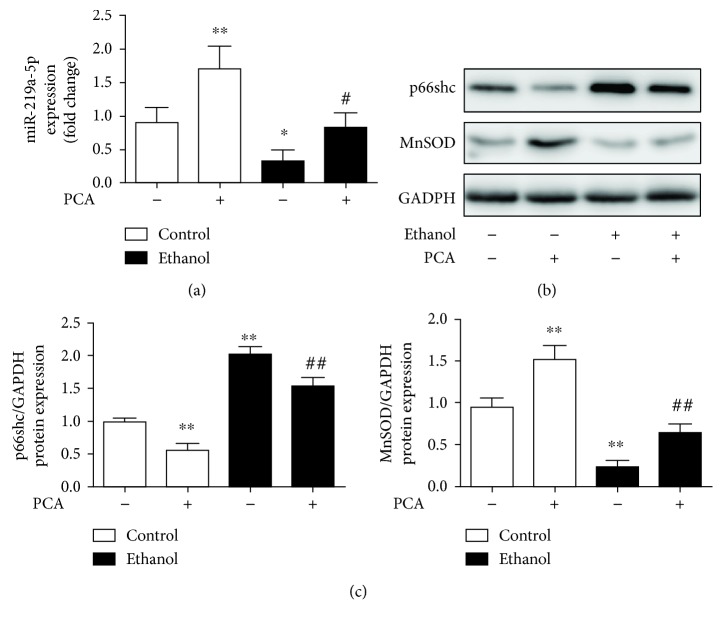
PCA exerted an antioxidant effect through the miR-219a-5p/p66shc pathway in ethanol-exposed primary hepatocytes. (a) qRT-PCR analysis of the miR-219a-5p expression in primary hepatocytes (*n* = 6). (b, c) Protein levels of p66shc and MnSOD (*n* = 3). ^∗∗^ *P* < 0.01 and ^∗^ *P* < 0.05 compared with the control group; ^##^ *P* < 0.01 and ^#^ *P* < 0.05 compared with the ethanol group.

Also, we obtain similar results from the primary hepatocytes in rats (Figure 8). MiR-219a-5p was activated by PCA treatment to inhibit p66shc protein levels and increase MnSOD levels in primary hepatocytes with ethanol exposure. Taken together, the results indicated that PCA at least partially decreased p66shc expression levels and ROS formation in a miR-219a-5p-dependent manner.

## 4. Discussion

Chronic alcoholic liver disease is widely identified as an important contributor to clinical morbidity; therefore, efficient and appropriate therapeutic agents and insight into the molecular mechanisms are necessary for the development of potential treatments. This the first study to demonstrate the following: (1) PCA contributes to the protection against ALD by reducing ROS formation and hepatocellular injury, (2) the miR-219a-5p/p66shc pathway is involved in ALD, and (3) the protective and antioxidant effect of PCA is mainly related to the modulation of the miR-219a-5p/p66shc pathway.

Protocatechuic acid (PCA), a compound that is commonly present in teas, vegetables, fruits, and many Chinese herbal medicines, is generally accepted as an antioxidant and anticarcinogenic agent [32]. In recent years, PCA has reportedly ameliorated chronic liver diseases and has been shown to protect against intestine and lung injuries [21, 22]. Also, PCA has been recognized as an efficient regulator of lipid profiles and oxidative stress [23, 24]. However, whether PCA treatment attenuates ALD remains unclear. In this study, we generated a Lieber-DeCarli diet-intake ALD model, and for the first time, we found that ALD was suppressed by PCA treatment, as indicated by the decrease in the serum index levels and the improvement of liver pathology. Moreover, we revealed the functional mechanisms through which PCA protects against ALD.

The p66shc molecule, an intracellular mediator, is related to ROS formation [22–24, 28]. p66Shc functions as an inducible redox enzyme, which is activated by stress and generates hydrogen peroxide (H_2_O_2_) [10, 33]. The deletion of p66shc in cells and tissues results in reduced levels of ROS and oxidative stress [34]. An increasing number of studies have shown that p66shc and downstream ROS formation are involved in ALD [1, 8, 10, 11]. Also, the generation and elimination of ROS is a dynamic equilibrium process [35, 36]. Ethanol exposure induces oxidative stress and stimulates ROS generation, which breaks the dynamic equilibrium balance and results in liver injury [37, 38]. In this study, p66shc expression assayed by western blotting, immunohistochemistry, and immunofluorescence analysis was increased in ALD and greatly reduced by the antioxidant PCA treatment. Moreover, p66shc-mediated ROS formation, indicated by DCFH-DA staining and H_2_O_2_ levels (Figures 3(g) and 3(h) and Figures 7(g) and 7(h)), was attenuated in ALD after PCA treatment. These data indicate that the PCA-mediated protection against ALD includes the downregulation of the p66shc expression and a reduction in downstream ROS formation.

miRNAs are potent gene regulators that have been associated with multiple pathophysiological processes [12, 14, 15]. Recently, it has been reported that miRNAs regulate the key pathogenic events and effect on ALD [17–19]. However, few reports have examined whether miRNAs mitigate ALD by reducing ROS formation. In this study, p66shc was predicted to be a potential target gene of miR-219a-5p with bioinformatics analysis, which was further determined in vivo and in vitro. And the results of the luciferase reporter assay showed that p66shc is a downstream target gene of miR-219a-5p. The miR-219a-5p binding sequences in the p66shc 3′-UTR are highly conserved across various species (Figure 4(c)). Studies regarding the silencing and overexpression of miRNA demonstrated that miR-219a-5p directly repressed the p66shc expression. These data revealed that miR-219a-5p might play an indispensable role in the control of the p66shc expression.

Consistently, the in vivo experiments revealed that PCA treatment could significantly alleviate Lieber-DeCarli diet-intake ALD and reverse the suppression of miR-219a-5p. Therefore, we hypothesized that the PCA-induced protection against ALD was related to the induction of miR-219a-5p. We then investigated this assumption in vitro and found that the miR-219a-5p expression was increased in PCA-treated AML-12 cells. Additionally, a decreased p66shc expression was observed in the ago-219a group compared with the control group. miR-219a-5p silencing reversed the PCA-mediated suppression of the p66shc expression and downstream ROS formation. Therefore, PCA stimulates the miR-219a-5p expression to inhibit the p66shc expression and downstream ROS formation, thereby attenuating alcoholic liver injury.

In summary, our results demonstrated for the first time that PCA could be a promising therapeutic agent for ALD via the miR-219a-5p/p66shc signaling pathway, which reduces ROS formation. The antioxidant functions of PCA treatment require further investigation, and this study may provide a new miRNA-based strategy for the treatment of alcoholic liver disease.

## Figures and Tables

**Table 1:** The sequences of siRNAs, agomirs, and antagomirs.

Name	Sequences (5′-3′)
p66shc-targeting siRNA	GCUGCAUCCCAACGACAAATT
	UUUGUCGUUGGGAUGCAGCTT
Negative control siRNA	UUCUCCGAACGUGUCACGUTT
	ACGUGACACGUUCGGAGAATT
miR-219a-5p-targeting agomir	UGAUUGUCCAAACGCAAUUCU
	AAUUGCGUUUGGACAAUCAUU
Negative control agomir	UUCUCCGAACGUGUCACGUTT
	ACGUGACACGUUCGGAGAATT
miR-219a-5p-targeting antagomir	AGAAUUGCGUUUGGACAAUCA
Negative control antagomir	CAGUACUUUUGUGUAGUACAA

**Table 2:** miRNAs are reported to be downregulated in ALD, and based on the TargetScan database, certain miRNAs may target p66shc.

Aberrantly expressed miRNAs in ALD					
Aberrantly expressed miRNAs	miR-21	miR-27a	miR-34a	miR-122	miR-125
	miR-126	miR-155	miR-182	miR-183	miR-199a
	miR-200a	miR-214	miR-217	miR-219	miR-320
	miR-375	miR-486	miR-513	miR-571	miR-652
	miR-705	miR-1224	let-7b		
Decreased miRNAs	miR-125	miR-126	miR-182	miR-183	miR-199a
	miR-200a	miR-214	miR-219	miR-652	let-7b
TargetScan	miR-183	miR-214	miR-219	let-7b	
Conservation	miR-183-5p.2	miR-219a-5p			

**Table 3:** qRT-PCR analysis of miRNAs in Lieber-DeCarli diet-induced alcoholic liver injury in rats (*n* = 6).

miRNAs	Fold change
miR-183-5p.2	0.374 ± 0.003^∗^
miR-219a-5p	0.425±0.012^∗∗^

^∗∗^ *P* < 0.01 and ^∗^ *P* < 0.05 compared with ND. Values are the mean ± SD, *n* = 6.

**Table 4:** qRT-PCR analysis of miRNAs in ethanol-exposed AML-12 cells (*n* = 6).

miRNAs	Fold change
miR-183-5p.2	Undetectable
miR-219a-5p	0.409±0.069^∗∗^

^∗∗^ *P* < 0.01 compared with the control group. Values are the mean ± SD, *n* = 6.

## Data Availability

The data used to support the findings of this study are included within the article.

## Abbreviations

PCA: Protocatechuic acid
ALD: Alcoholic liver disease
ROS: Reactive oxygen species
ALT: Alanine aminotransferase
AST: Aspartate aminotransferase
TC: Total cholesterol
TG: Triglyceride
H&E staining: Hematoxylin and eosin staining
MDA: Malondialdehyde
CAT: Catalase
GSH: Glutathione
MnSOD: Manganese superoxide dismutase
DCFH-DA: 2′,7′-Dichlorodihydrofluorescein diacetate.

## Supplementary Materials

Supplementary Materials Figure S1: AML-12 cells were grown in 96-well plates, and after being exposed with 0, 25, 50, 100, and 200 mM ethanol for 24 h, the cells were incubated with a CCK-8 assay. The absorbance of each individual well was determined at 570 nm (Supplementary Figure 1A). Subsequently, AML-12 cells were grown in 96-well plates and pretreated with 0, 1.25, 2.5, 5, 10, and 20 *μ*M PCA for 6 h and 100 mM ethanol for 24 h. Then, the cells were incubated with a CCK-8 assay, and the absorbance of each individual well was determined at 570 nm (Supplementary Figure 1B). Figure S2: graphic abstract. Figure S3: AML-12 cells were transfected with ago-219a-5p, ago-219a-2-3p, and ago-219b-5p. Ago-NC was used as a negative control, and the p66shc expression was determined. Figure S4: AML-12 cells were incubated with an ADH inhibitor (fomepizole (4-methylpyrazole or 4-MP), 10 *μ*m, Selleckchem, Houston, TX, USA) or an ALDH inhibitor (disulfiram (DSF), 10 *μ*m, Selleckchem, Houston, TX, USA) for 24 h and exposed with ethanol for 24 h. miR-219a-5p and p66shc expressions were determined.

## References

[1] Liangpunsakul S., Crabb D. W. (2016). Early detection of alcoholic liver disease: are we a step closer?. *Gastroenterology*.

[2] Rehm J., Samokhvalov A. V., Shield K. D. (2013). Global burden of alcoholic liver diseases. *Journal of Hepatology*.

[3] Pavlov C. S., Casazza G., Semenistaia M. (2016). Ultrasonography for diagnosis of alcoholic cirrhosis in people with alcoholic liver disease. *Cochrane Database of Systematic Reviews*.

[4] Szabo G. (2015). Gut-liver axis in alcoholic liver disease. *Gastroenterology*.

[5] Thiele M., Detlefsen S., Sevelsted Møller L. (2016). Transient and 2-dimensional shear-wave elastography provide comparable assessment of alcoholic liver fibrosis and cirrhosis. *Gastroenterology*.

[6] Ceni E., Mello T., Galli A. (2014). Pathogenesis of alcoholic liver disease: role of oxidative metabolism. *World Journal of Gastroenterology*.

[7] Leung T. M., Nieto N. (2013). CYP2E1 and oxidant stress in alcoholic and non-alcoholic fatty liver disease. *Journal of Hepatology*.

[8] Heinrich J. N., Kwak S. P., Howland D. S. (2006). Disruption of ShcA signaling halts cell proliferation--characterization of ShcC residues that influence signaling pathways using yeast. *Cellular Signalling*.

[9] Almeida M., Han L., Ambrogini E., Bartell S. M., Manolagas S. C. (2010). Oxidative Stress Stimulates Apoptosis and Activates NF-*κ*B in Osteoblastic Cells via a PKC*β*/p66shcSignaling Cascade: Counter Regulation by Estrogens or Androgens. *Molecular Endocrinology*.

[10] Berniakovich I., Trinei M., Stendardo M. (2008). p66Shc-generated oxidative signal promotes fat accumulation. *The Journal of Biological Chemistry*.

[11] Haga S., Terui K., Fukai M. (2008). Preventing hypoxia/reoxygenation damage to hepatocytes by p66shc ablation: Up-regulation of anti-oxidant and anti-apoptotic proteins. *Journal of Hepatology*.

[12] Derdak Z., Villegas K. A., Wands J. R. (2012). Early growth response-1 transcription factor promotes hepatic fibrosis and steatosis in long-term ethanol-fed Long-Evans rats. *Liver International*.

[13] Koch O. R., Fusco S., Ranieri S. C. (2008). Role of the life span determinant P66shcA in ethanol-induced liver damage. *Laboratory Investigation*.

[14] Shukla G. C., Singh J., Barik S. (2011). MicroRNAs: processing, maturation, target recognition and regulatory functions. *Molecular and Cellular Pharmacology*.

[15] Neilson J. R., Sharp P. A. (2008). Small RNA regulators of gene expression. *Cell*.

[16] Pirola C. J., Fernández Gianotti T., Castaño G. O. (2015). Circulating microRNA signature in non-alcoholic fatty liver disease: from serum non-coding RNAs to liver histology and disease pathogenesis. *Gut*.

[17] Li Q., Kim Y. R., Vikram A. (2016). P66Shc-induced microRNA-34a causes diabetic endothelial dysfunction by downregulating sirtuin1. *Arteriosclerosis, Thrombosis, and Vascular Biology*.

[18] McDaniel K., Herrera L., Zhou T. (2014). The functional role of microRNAs in alcoholic liver injury. *Journal of Cellular and Molecular Medicine*.

[19] Momen-Heravi F., Bala S., Kodys K., Szabo G. (2015). Exosomes derived from alcohol-treated hepatocytes horizontally transfer liver specific miRNA-122 and sensitize monocytes to LPS. *Scientific Reports*.

[20] Saha B., Bruneau J. C., Kodys K., Szabo G. (2015). Alcohol-induced miR-27a regulates differentiation and M2 macrophage polarization of normal human monocytes. *Journal of Immunology*.

[21] Ma L., Wang G., Chen Z. (2014). Modulating the p66shc signaling pathway with protocatechuic acid protects the intestine from ischemia-reperfusion injury and alleviates secondary liver damage. *The Scientific World Journal*.

[22] Wang G. Z., Yao J. H., Jing H. R. (2012). Suppression of the p66shc adapter protein by protocatechuic acid prevents the development of lung injury induced by intestinal ischemia reperfusion in mice. *Journal of Trauma and Acute Care Surgery*.

[23] Krajka-Kuźniak V., Szaefer H., Baer-Dubowska W. (2008). Hepatic and extrahepatic expression of glutathione S-transferase isozymes in mice and its modulation by naturally occurring phenolic acids. *Environmental Toxicology and Pharmacology*.

[24] Lane J. S., Todd K. E., Lewis M. P. N. (1997). Interleukin-10 reduces the systemic inflammatory response in a murine model of intestinal ischemia/reperfusion. *Surgery*.

[25] Adefegha S. A., Omojokun O. S., Oboh G. (2015). Modulatory effect of protocatechuic acid on cadmium induced nephrotoxicity and hepatoxicity in rats in vivo. *Springerplus*.

[26] HUR J. M., PARK J. G., YANG K. H. (2003). Effect of methanol extract of Zanthoxylum piperitum leaves and of its compound, protocatechuic acid, on hepatic drug metabolizing enzymes and lipid peroxidation in rats. *Bioscience, Biotechnology, and Biochemistry*.

[27] Seglen P. O. (1976). Chapter 4 Preparation of Isolated Rat Liver Cells. *Methods in Cell Biology*.

[28] Gao L., Shan W., Zeng W. (2016). Carnosic acid alleviates chronic alcoholic liver injury by regulating the SIRT1/ChREBP and SIRT1/p66shc pathways in rats. *Molecular Nutrition & Food Research*.

[29] Dippold R. P., Vadigepalli R., Gonye G. E., Patra B., Hoek J. B. (2013). Chronic ethanol feeding alters miRNA expression dynamics during liver regeneration. *Alcoholism, Clinical and Experimental Research*.

[30] Dong X., Liu H., Chen F., Li D., Zhao Y. (2014). MiR-214 promotes the alcohol-induced oxidative stress via down-regulation of glutathione reductase and cytochrome P450 oxidoreductase in liver cells. *Alcoholism, Clinical and Experimental Research*.

[31] Tang Y., Zhang L., Forsyth C. B., Shaikh M., Song S., Keshavarzian A. (2015). The role of miR-212 and iNOS in alcohol-induced intestinal barrier dysfunction and steatohepatitis. *Alcoholism, Clinical and Experimental Research*.

[32] Chan K., Chui S. H., Wong D. Y. L., Ha W. Y., Chan C. L., Wong R. N. S. (2004). Protective effects of danshensu from the aqueous extract of Salvia miltiorrhiza (Danshen) against homocysteine-induced endothelial dysfunction. *Life Sciences*.

[33] Giorgio M., Migliaccio E., Orsini F. (2005). Electron transfer between cytochrome c and p66Shc generates reactive oxygen species that trigger mitochondrial apoptosis. *Cell*.

[34] Beltrami E., Valtorta S., Moresco R. (2013). The p53-p66Shc apoptotic pathway is dispensable for tumor suppression whereas the p66Shc-generated oxidative stress initiates tumorigenesis. *Current Pharmaceutical Design*.

[35] Vohr H. (2005). Oxidative stress.

[36] Li S., Tan H. Y., Wang N. (2015). The role of oxidative stress and antioxidants in liver diseases. *International Journal of Molecular Sciences*.

[37] Huang Q.-H., Xu L.-Q., Liu Y.-H. (2017). Polydatin Protects Rat Liver against Ethanol-Induced Injury: Involvement of CYP2E1/ROS/Nrf2 and TLR4/NF-*κ*B p65 Pathway. *Evidence-based Complementary and Alternative Medicine*.

[38] Zhou L., Peng Y., Wang Q., Lin Q. (2017). An ESIPT-based two-photon fluorescent probe detection of hydrogen peroxide in live cells and tissues. *Journal of Photochemistry and Photobiology. B*.

