# Supplementary material for: Protocatechuic Acid-Mediated miR-219a-5p Activation Inhibits the p66shc Oxidant Pathway to Alleviate Alcoholic Liver Injury
Source: Oxid Med Cell Longev. 2019 Jul 24;2019:3527809. doi: 10.1155/2019/3527809 (PMC6683775; doi:10.1155/2019/3527809)
Supplement: Supplementary Materials — Figure S1: AML-12 cells were grown in 96-well plates, and after being exposed with 0, 25, 50, 100, and 200 mM ethanol for 24 h, the cells were incubated with a CCK-8 assay. The absorbance of each individual well was determined at 570 nm (Supplementary Figure 1A). Subsequently, AML-12 cells were grown in 96-well plates and pretreated with 0, 1.25, 2.5, 5, 10, and 20 μM PCA for 6 h and 100 mM ethanol for 24 h. Then, the cells were incubated with a CCK-8 assay, and the absorbance of each individual well was determined at 570 nm (Supplementary Figure 1B). Figure S2: graphic abstract. Figure S3: AML-12 cells were transfected with ago-219a-5p, ago-219a-2-3p, and ago-219b-5p. Ago-NC was used as a negative control, and the p66shc expression was determined. Figure S4: AML-12 cells were incubated with an ADH inhibitor (fomepizole (4-methylpyrazole or 4-MP), 10 μm, Selleckchem, Houston, TX, USA) or an ALDH inhibitor (disulfiram (DSF), 10 μm, Selleckchem, Houston, TX, USA) for 24 h and exposed with ethanol for 24 h. miR-219a-5p and p66shc expressions were determined. [file 3527809.f1.pdf]

Supplementary Figures.

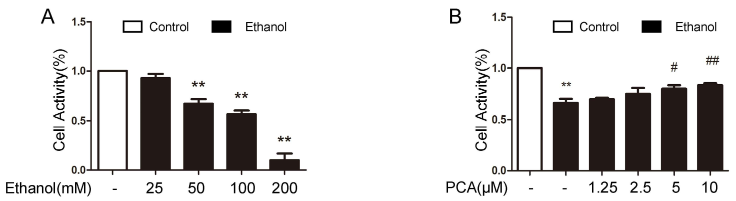

Figure S1. The protective effects of PCA against ethanol-exposed injury in AML12 cells. (A) AML-12 cell viability with 0, 25, 50, 100, and 200 mM ethanol exposure for 24 h. (B) AML-12 cell viability after 0, 1.25, 2.5, 5, 10, 20 μM PCA treatment for 6 h and 100 mM ethanol for 24 h. **P<0.01 and *P<0.05 compared with the Control group; $^{\#\#}$P<0.01 and $^{\#}$P<0.05 compared with the Ethanol group.

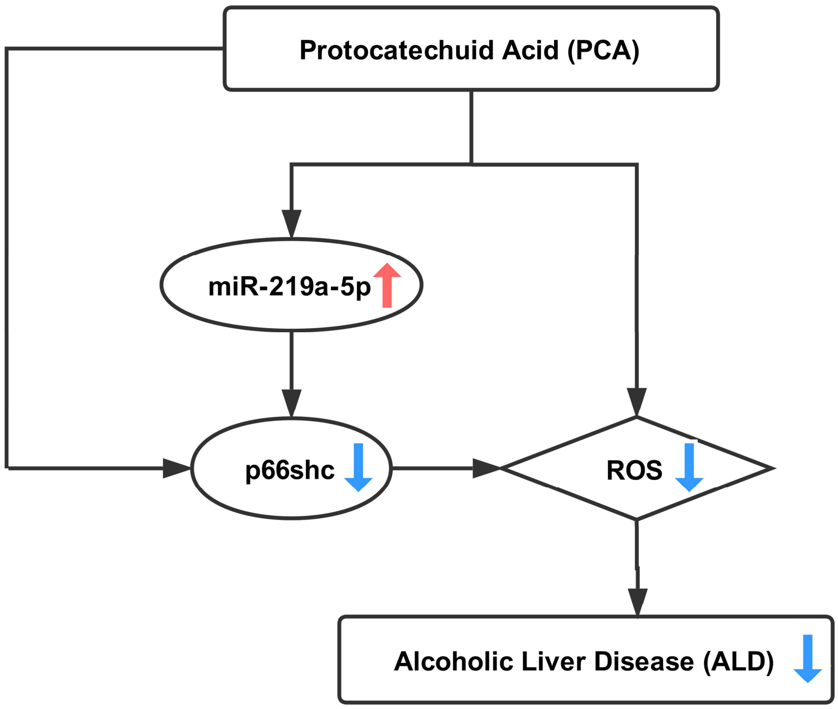

Figure S2. Graphic abstract.

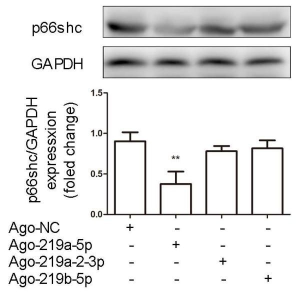

Figure S3. AML-12 cells were transfected with ago-219a-5p, ago-219a-2-3p, ago-219b-5p. Ago-NC was used as a negative control. P66shc protein levels were determined by western blotting (n=3). $^{**}P<0.01$ compared with ago-NC.

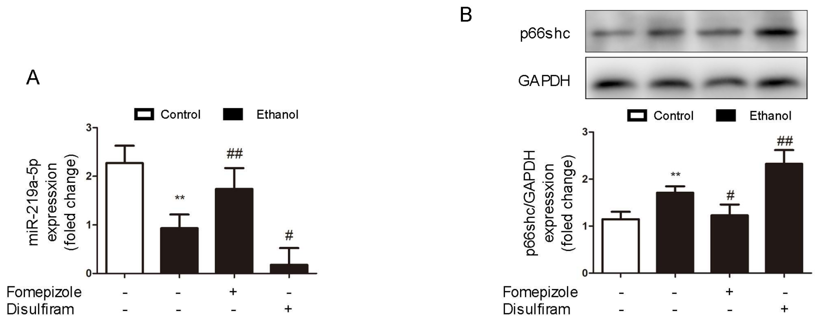

Figure S4. AML-12 cells were treated with fomepizole and disulfiram before ethanol exposure, and miR-219a-5p and p66shc expression were determined. (A) miR-219a-5p expression (n=6). (B) p66shc expression (n=3). **P<0.01 compared with the Control group; $^{\#\#}$P<0.01 and $^{\#}$P<0.05 compared with the Ethanol group.
